# Incidence and impact of pseudoprogression and mixed responses in metastatic renal cell carcinoma patients treated with ipilimumab/nivolumab: a retrospective analysis

**DOI:** 10.2340/ao.v65.45460

**Published:** 2026-05-06

**Authors:** Aaron Caeyman, Giulia Mammone, Lisa Kinget, Marcella Baldewijns, Liesbeth De Wever, Maarten Albersen, Philip R. Debruyne, Octavie Demeulenaere, Edward Scott McTaggart, Saurabh Saraswat, Charlien Berghen, Gert De Meerleer, Stefan Naulaerts, Abhishek D. Garg, Benoit Beuselinck

**Affiliations:** a Department of General Medical Oncology, Leuven Cancer Institute, University Hospitals Leuven, Leuven, Belgium; b Department of Pathology, University Hospitals Leuven, Leuven, Belgium; c Department of Radiology, University Hospitals Leuven, Leuven, Belgium; d Department of Urology, University Hospitals Leuven, Leuven, Belgium; e Medical Oncology Department, Kortrijk Cancer Centre, az groeninge, Kortrijk, Belgium; f Medical Technology Research Centre (MTRC), School of Life Sciences, Anglia Ruskin University, Cambridge, UK; g School of Nursing & Midwifery, University of Plymouth, Plymouth, UK; h Laboratory of Cell Stress & Immunity (CSI), Department of Cellular & Molecular Medicine, KU Leuven, Leuven, Belgium; i Department of Radiotherapy/Oncology, University Hospitals Leuven, Leuven, Belgium; j Laboratory of Computational Oncology, Department of Oncology, KU Leuven, Leuven, Belgium

**Keywords:** immune checkpoint inhibitor, atypical response, treatment beyond progression, iRECIST, kidney cancer

## Abstract

**Introduction:**

Immune checkpoint inhibitors can elicit atypical responses such as pseudoprogression (psPD) and mixed responses (MR). We aimed to analyze the occurrence and prognostic impact of atypical responses in metastatic clear-cell renal cell carcinoma (m-ccRCC) patients treated with ipilimumab/nivolumab.

**Materials and methods:**

In this retrospective series of m-ccRCC patients treated with ipilimumab/nivolumab in first-line, we performed radiographic evaluation using Response Evaluation Criteria in Solid Tumors and iRECIST consensus guidelines and compared both methods.

**Results:**

We assessed 258 baseline target lesions in 100 eligible patients. MR occurred in 24% of patients. In 15 patients (62%), the MR evolved to confirmed progressive disease (cPD), while in nine patients (38%), the MR evolved toward a partial response (PR) and was thus a psPD. psPD occurred in 13% of patients: with increase of all lesions in four patients or with MR features in nine patients. psPD patients had the second best time-to-progression (TTP) and cancer-specific survival (CSS), slightly lower compared to TTP and CSS in patients with PR without a phase of psPD and better than TTP and CSS in patients with stable disease as best response. From all patients who presented with PD at first CT evaluation (*n* = 40), including 17 patients with unconfirmed progressive disease and 23 patients with MR, in 12 (30%) patients this was a psPD and led to a PR.

**Conclusions:**

Atypical responses such as psPD and MR occurred in 13 and 24% of m-ccRCC patients treated with ipilimumab/nivolumab. Patients with psPD had favorable outcomes, while MR in most cases evolved to progression.

## MICROABSTRACT

Outcome of metastatic renal cell carcinoma (RCC) has significantly improved with immune checkpoint inhibitors (ICI). However, patients have atypical responses such as mixed response and pseudoprogression. We retrospectively analyzed 100 RCC patients and found 13% of pseudoprogressors with outcome only slightly inferior to outcome in responders. Our study adds to the limited evidence in this area, helping clinicians treat their patients with ICI.

**CLINICAL PRACTICE POINTS**

According to our data, pseudoprogression (psPD), as defined by iRECIST, occurs in around 13% of patients diagnosed with metastatic clear-cell renal cell carcinoma (m-ccRCC) and treated with ipilimumab/nivolumab in first-line. psPD can occur at first or second computed tomography (CT) evaluation and is associated with favorable outcome, similar to outcome in patients with partial response (PR) as best response who did not experience psPD.

Mixed responses (MR) occurred in 24% of m-ccRCC patients treated with ipilimumab/nivolumab, with most cases (62%) being real progressors.

In case of unconfirmed progressive disease (uPD) or MR at first evaluation, three out of 10 patients did not evolve to cPD, but to PR or to disease stabilization.

Roughly one out of four patients who eventually reached a PR on ipilimumab/nivolumab, experienced psPD.

Hence, it is important to continue ipilimumab/nivolumab (or nivolumab monotherapy after four cycles of ipilimumab/nivolumab combination) until the second CT evaluation in case of uPD or a MR at first CT evaluation.

## Introduction

Renal cell carcinoma (RCC) is a malignant tumor originating from the renal tubular epithelial cells, accounting for a comprehensive 80% of all primary kidney tumors [1]. About 430,000 new patients were diagnosed with RCC in 2022. There were about 150,000 RCC-related deaths, with RCC ranking as the 16th most common cause of cancer death worldwide [2].

In the last two decades, several new treatment options have become available, resulting in improved outcomes for these patients [3]. One of the most significant treatment improvements was the introduction of immune checkpoint inhibitors (ICI), such as nivolumab, pembrolizumab and ipilimumab. In 2015, the phase III trial with nivolumab, a fully human antibody targeting programmed cell death protein 1 (PD-1), was published. Results from this trial showed increased overall survival (OS) and less severe toxicity compared to mammalian target of rapamycin-mediated pathways targeted therapy (everolimus) in patients with metastatic clear-cell renal cell carcinoma (m-ccRCC) pre-treated with vascular endothelial growth factor receptor tyrosine kinase inhibitors (VEGFR-TKI) [4]. Further pivotal trials introduced combined ICI treatments with nivolumab and ipilimumab (an anti–cytotoxic T-lymphocyte–associated antigen four antibody) as well as the combination of VEGFR-TKI and ICI in the first-line treatment of m-ccRCC [5–9].

According to the most recent American Society of Clinical Oncology and European Society of Medical Oncology guidelines either combined ICI or concurrent ICI and VEGFR-TKI treatment are recommended in first-line setting, depending on risk stratification and patient profile. In select cases, monotherapy with either VEGFR-TKI or PD-1 ICI can be considered [10–12].

The introduction of ICI has complicated the evaluation of response in the different cancer types where this treatment is being used. Traditionally, the criteria defined by the Response Evaluation Criteria in Solid Tumors (RECIST) Working Group have been widely used to evaluate tumor response. However, in several tumor types treated with ICIs, patients treated with ICI have shown transient tumor size increases or appearance of new lesions due to immune cell infiltrates and edema followed by subsequent regression or stabilization. This phenomenon, called pseudoprogression (psPD), was first described during the treatment of melanoma with ipilimumab, and has subsequently also been documented during ICI treatment in m-ccRCC and non-small cell lung carcinoma (NSCLC) [13–15]. The discovery of psPD was one of the driving factors for the RECIST group to introduce revised criteria in order to better evaluate response in patients treated with ICI. These new criteria, published in 2017 as iRECIST, have changed the definition of progressive disease (PD) by introducing the concept of immune unconfirmed and confirmed progressive disease (uPD and cPD) [16]. psPD has been associated with favorable outcome on ICI in multiple tumor types, such as melanoma and NSCLC [17, 18]. Another atypical response pattern observed with ICI, is mixed response (MR), defined as the increase of some lesions and response of other lesions, also observed in NSCLC [17].

Our team was the first to publish data on psPD and MR in m-ccRCC patients treated with nivolumab. Our retrospective analysis of m-ccRCC treated with nivolumab monotherapy showed the incidence of atypical responses such as psPD and MR occurred in 8.5 and 11.7% of the patients. In this study psPD was associated with favorable outcomes, while MR most often evolved to progression [19].

However, the incidence and impact of psPD in m-ccRCC patients treated with ipilimumab/nivolumab is unknown. Therefore, we aimed to analyze our own cohort of m-ccRCC patients treated with ipilimumab/nivolumab in combination therapy in order to detect the incidence and outcome of atypical response patterns such as psPD and MR.

## Patients and methods

We retrospectively reviewed the records of all patients in our database with m-ccRCC who started on intravenous combination therapy of ipilimumab (1 mg/kg every 3 weeks) and nivolumab (3 mg/kg every 3 weeks) for four cycles, followed by nivolumab in monotherapy (240 mg fixed dose every 2 weeks and/or 480 mg fixed dose every 4 weeks) in first-line between January 30th 2015 and May 7th 2024 at the University Hospitals Leuven and the general hospital AZ Groeninge in Belgium. All patients were treated in routine clinical practice. Timing and schedule were left to the discretion of the attending doctors in accordance with applicable local and international practice guidelines at time of treatment. All patients had previously provided written informed consent to participate in this database and ensuing analyses. The study was conducted in accordance with the Declaration of Helsinki. This study was approved by the respective hospital ethics committees (S63833).

Patient data were collected at diagnosis of kidney cancer (age, gender, synchronous metastases, sarcomatoid dedifferentiation) and at start of ipilimumab-nivolumab (age, Eastern Cooperative Oncology Group (ECOG) performance status, International Metastatic Renal Cell Carcinoma Database Consortium (IMDC) prognostic score [20], number and sites of metastases, tumor burden (as calculated by the sum of the largest diameter of the target lesions), laboratory findings and prior systemic therapy). Data on time-to-progression (TTP) and cancer specific survival (CSS) were collected. TTP was preferred above progression-free survival (PFS) and CSS above OS, because in patients with durable responses, death nowadays occur more often for non-cancer-related causes. We also collected data on tumor shrinkage in order to extensively illustrate the impact of atypical responses.

All patients on ipilimumab/nivolumab underwent thoracic and abdominal computed tomography (CT) every 2–3 months, as well as brain imaging (CT and/or magnetic resonance imaging) on indication. In the participating hospitals, the first CT after start of ipilimumab/nivolumab is usually performed after 9–12 weeks of therapy. The second or confirmatory CT is usually performed 9–12 weeks later. All available imaging was reviewed for tumor response and interpreted according to the latest RECIST and iRECIST criteria [16]. We compared both response assessment methods.

Exclusion criteria were as follows: By iRECIST, a confirmatory second CT is necessary in order to confirm uPD at first CT. Without second CT, it is impossible to know if a uPD or a MR on the first CT will evolve toward cPD or a partial response (PR). Hence, patients who only had one radiographic evaluation under ipilimumab/nivolumab were excluded from our analysis. Patients with lesions difficult to measure (for instance peritoneal infiltration) were also excluded. Patients who underwent radiotherapy or surgery for an unconfirmed progressing target lesion were excluded from further analysis, as subsequent treatment on ipilimumab/nivolumab no longer allowed determination of whether the lesion would have progressed or regressed. Cut-off for all data collection was on November 1st 2025.

We defined two groups of atypical responses based on the first two radiographic evaluations: psPD and MR. psPD was defined as patients who achieved uPD at first (CT1) and/or second (CT2) radiographic evaluation, later followed by either stable disease (SD), partial or complete response (PR or CR). This could only be detected and defined a posteriori. MR was defined as the presence of PD in one or more (non-)target lesions with PR in at least one (non-)target lesion. Consequently, patients classified as having MR could have an iRECIST evaluation ranging from PR to uPD. Over time, MR could evolve into cPD or into clinical benefit (SD or PR), the latter being classified as psPD.

Percentages were compared with Fisher’s exact test. One-way ANOVA was performed to compare means of different groups. Time-to-event analyses were done with Kaplan-Meier estimates and compared with the log-rank test. All statistical analyses were conducted using the GraphPad Prism 10 software (GraphPad Software, LLC).

## Results

### Included patients and patients’ characteristics

**Figure 1:**
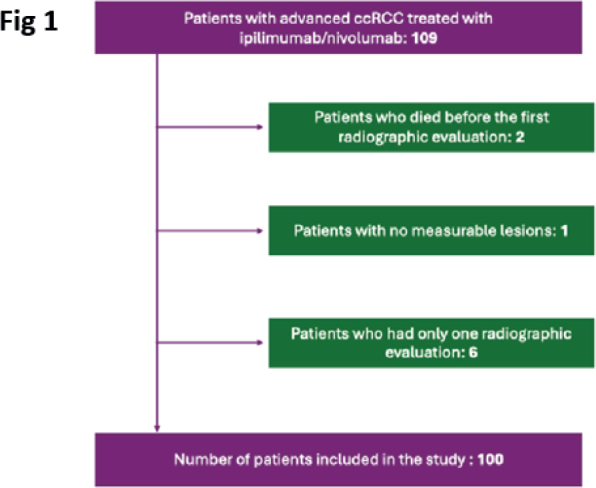
Flowchart describing exclusion of patients. ccRCC: clear-cell renal cell carcinoma.

Across the two participating centers, 109 patients with available imaging were screened for inclusion in our analysis. Nine patients were excluded from the final analysis (Flowchart in Figure 1). Two patients died before the first radiographic evaluation under ipilimumab/nivolumab could be performed. One patient had no measurable lesions. Five patients had only one radiographic evaluation under ICI treatment, all of these showed early uPD, making it impossible to exclude psPD. One patient had only a thoracic CT performed as first evaluation, but no abdominal CT. Hence, complete response assessment was not possible. Thus, our final analysis included 100 patients.

Patients’ characteristics are shown in Table 1. In our cohort, the median age of all patients at start of ICI was 66 years (range, 42–90 years) and 73% were male. ECOG Performance Status was 0 in 74% of patients and 1 in 26% of patients. A total of 15 patients had good, 64 intermediate and 21 poor IMDC prognostic risk. A total of 77% of patients had prior nephrectomy, no patients received prior treatment with VEGFR-TKI.

**Table 1:** Patients’ characteristics of different response groups, based on response pattern during first four radiographic evaluations.

Characteristics		Full cohort (*n* = 100)	PR without psPD (*n* = 33)	SD (*n* = 26)	uPD > cPD (*n* = 14)	MR > cPD (*n* = 14)	psPD (*n* = 13)
Median age in years at start ICI (range)		66 (42–90)	67 (42–90)	65.5 (48–79)	62.5 (48–80)	70 (46–79)	61 (44–80)
Male sex, *n* (%)		73/100 (73%)	24/33 (73%)	19/26 (73%)	10/14 (71%)	8/14 (57%)	12/13 (92%)
Sarcomatoid dedifferentiation present, *n* (%)		*15/64 (23%)*	5/24 (21%)	4/16 (25%)	1/6 (17%)	1/8 (13%)	4/10 (40%)
Sarcomatoid component compared to total tumor (mean)		5%	7%	2%	1%	0%	8%
IMDC Risk group, *n* (%)	Good	15/100 (15%)	7/33 (21%)	5/26 (19%)	/	1/14 (7%)	2/13 (15%)
	Intermediate	64/100 (64%)	22/33 (66%)	14/26 (54%)	8/14 (57%)	9/14 (64%)	11/13 (85%)
	Poor	21/100 (21%)	4/33 (12%)	7/26 (27%)	6/14 (43%)	4/14 (29%)	/
ECOG PS	0	74/100 (74%)	29/33 (88%)	18/26 (69%)	8/14 (57%)	8/14 (57%)	11/13 (85%)
	1	26/100 (26%)	4/33 (12%)	8/26 (31%)	6/14 (43%)	6/14 (43%)	2/13 (15%)
Number of evaluable target lesions, *n* (%)	1	26/100 (26%)	13/33 (39%)	6/26 (23%)	3/14 (21%)	2/14 (14%)	1/13 (8%)
	2 or more	74/100 (74%)	20/33 (60%)	20/26 (77%)	11/14 (79%)	12/14 (86%)	12/13 (92%)
Tumor burden (median)(mm)		70	54	72	69	85	57

PR: partial response; SD: stable disease; uPD: unconfirmed progressive disease; cPD: confirmed progressive disease; MR: mixed response; psPD: pseudoprogression; ICI: immune checkpoint inhibitor; IMDC: International Metastatic Renal Cell Carcinoma Database Consortium; ECOG PS: Eastern Cooperative Oncology Group Performance Status.

Overall, 258 iRECIST evaluable baseline target lesions were assessed, with the median number of lesions per patient being 2 (range, 1–6). The most commonly assessed sites were lung (49%), lymph nodes (38%), adrenal gland (11%) and kidney (10%).

Global median TTP was 12 months and global median CSS 67 months. We observed 73 PFS-events, but only 70 TTP-events. A total of 51 patients died, but 13 for non-cancer-related reasons. Global median follow-up since start of ipilimumab/nivolumab was 50 months.

Timing of imaging was as follows: The median interval between start of ipilimumab/nivolumab and CT1 was 77 days (11 weeks) (interquartile range 23 days) and the median interval between CT1 and CT2 was 84 days (12 weeks) (interquartile range 29 days). CT1 was performed around 9 weeks (±10 days) in 36 patients and around 12 weeks (±10 days) in 45 patients). In 12 patients, CT1 was performed earlier (after a median of 46 days or 7.5 weeks) because of clinical need (suspicion of progression). In seven patients, CT1 was performed after 12 weeks (median around 14 weeks) mainly for planification issues. CT2 was performed around 9 weeks after CT1 (±10 days) in 14 patients and around 12 weeks after CT1 (±10 days) in 42 patients. In 16 patients, CT2 was performed earlier (after a median of 42 days or 7 weeks). In 27 patients, CT2 was performed after 12 weeks (median around 15 weeks).

### Response evolution and definition of cases of MR and psPD

**Figure 2:**
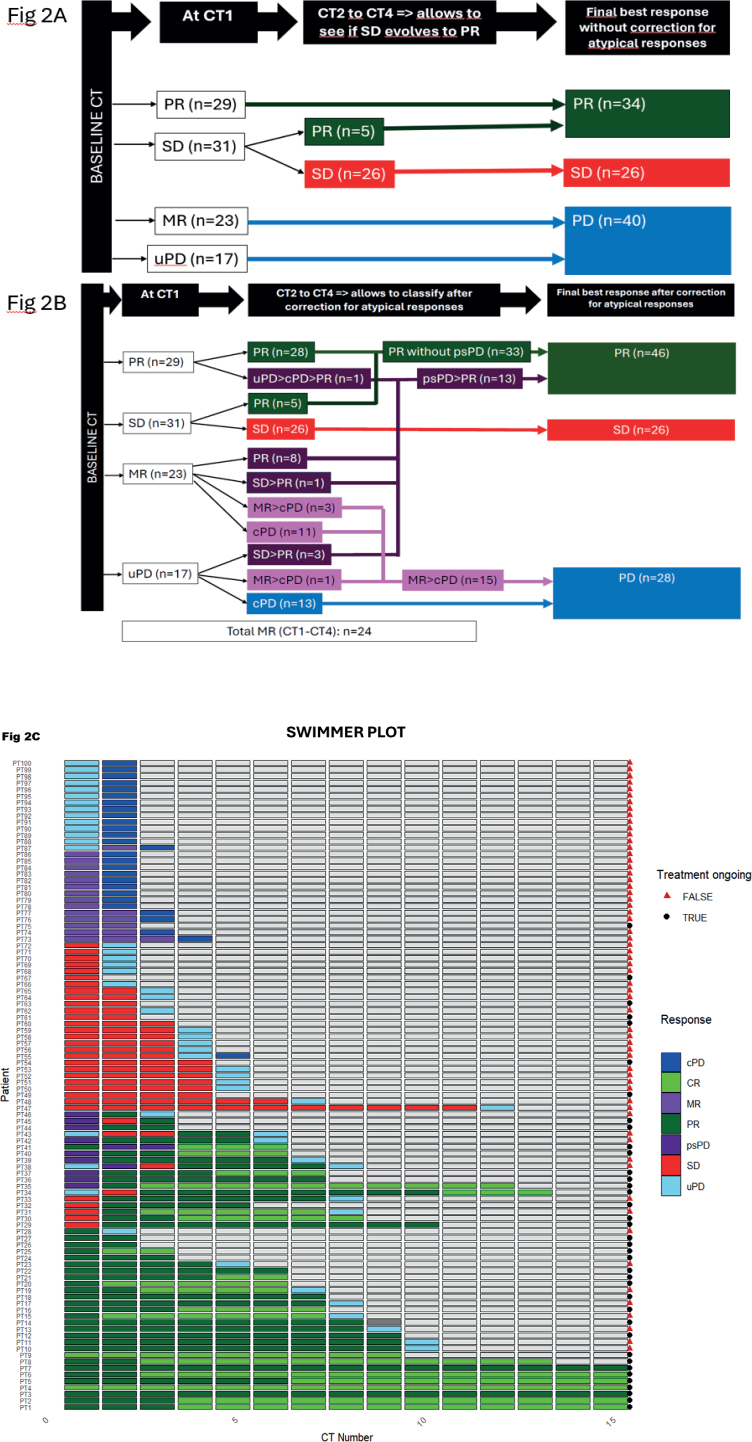
Response assessment from baseline CT to CT4 and final best response estimation. Panel A: with RECIST, without correction for atypical responses. Panel B: with iRECIST, allowing to detect atypical responses such as MR and psPD. In dark purple: patients displaying psPD. In light purple: patients displaying MR leading to PD. In green: patients with PR or CR. In red: patients with SD. In blue: patients displaying PD as best response. Panel C: swimmers plot. CT: computed tomography; PD: progressive disease; PR: partial response; SD: stable disease; MR: mixed response; uPD: unconfirmed progressive disease; cPD: confirmed progressive disease; psPD: pseudoprogression; CR: complete response.

Figure 2 shows the evolution of response from baseline CT to CT4: in panel A according to RECIST, in panel B according to iRECIST (including MR and psPD) and in panel C as a swimmers plot illustrating the evolution of response in each patient.

Best response following RECIST (Figure 2 panel A) was PR in 34 patients (34%) (among them 13 with CR), SD in 26 patients (26%) and PD in 40 patients (40%) (including all patients with PD or MR on CT1).

With iRECIST (Figure 2 panel B), after observing the evolution through CT2–CT4, we identified 24 (24%) MRs, among them 23 at CT1 and one at CT2. Fifteen (62%) MRs evolved toward a cPD, while 9 (38%) evolved toward a PR and could hence be classified as psPD. We identified four additional psPD patients, one on CT2 after PR on CT1 and 3 after uPD on CT1. Hence, the total number of psPD patients was 13 (13%). After correction for atypical responses, we divided our patient cohort in five categories: PR as best response without passing through psPD (*n* = 33), SD as best response (*n* = 26), uPD evolving to cPD as best response (*n* = 14), MR evolving to cPD as best response (*n* = 14) and psPD (*n* = 13). CR occurred in 17 patients: in 13 patients without psPD and in four patients after psPD. All the patients with psPD, eventually had PR as best response.

Hence, from all patients who presented with PD at CT1 following RECIST (*n* = 40), including 17 patients with uPD and 23 patients with MR, in 12 (30%) patients this was a psPD and led to a PR or CR. Hence, when a first CT scan evaluation shows uPD or MR, the patient still had three chances out of 10 to evolve toward a PR. From all the patients who eventually displayed PR/CR as best response (*n* = 46), 13 (28%) passed through psPD.

psPD was most often observed in the following host organs: adenopathies (*n* = 7), lung (*n* = 8) and bone (*n* = 6). We also observed it in kidney tumors (*n* = 5), liver (*n* = 3), peritoneum (*n* = 3), adrenal gland (*n* = 2), myocard (*n* = 1) and thyroid metastases (*n* = 1). We did not observe the phenomenon in skin, pleura, brain and pancreatic metastatic sites.

### Correlation of best response with TTP and CSS before correction for atypical responses

**Figure 3:**
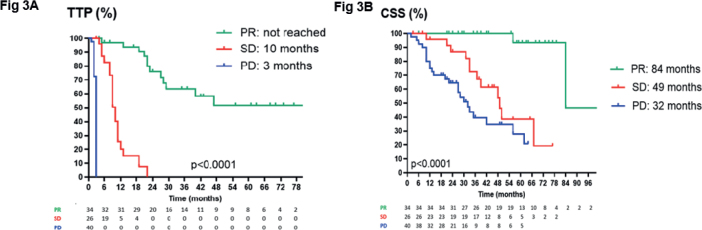
Kaplan–Meier estimates of TTP (PANEL A) and CSS (PANEL B) is shown in patients classified according to overall best response, based on RECIST response. Patients with psPD at first CT and patients with MR as best response at first CT are pooled with the PD patients. TTP: time to progression; CSS: cancer-specific survival; PR: partial response; SD: stable disease; PD: progressive disease; CT: computed tomography; MR: mixed response.

Figure 3 shows the outcome according to best RECIST response in terms of TTP and CSS. In responders, TTP was not reached and CSS 84 months. Patients with SD as best response obtained a TTP of 10 months and a CSS of 49 months. Patients with early PD reached a TTP of 3 months and a CSS of 32 months. Note that in patients with PD as best response, although median TTP was only 3 months, 8 patients out of 40 were still alive at 36 months.

### Correlation of best response with TTP and CSS after correction for atypical responses

**Figure 4:**
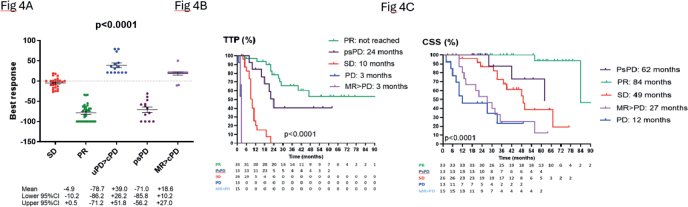
Maximal tumor shrinkage (PANEL A), Kaplan–Meier estimates of TTP (PANEL B) and Kaplan–Meier estimates of CSS (PANEL C) is shown in patients according to best iRECIST response and subdivision in 5 subgroups after correction for atypical responses. TTP: time to progression; CSS: cancer-specific survival; psPD: pseudoprogression; PR: partial response; SD: stable disease; MR: mixed response; uPD: unconfirmed progressive disease; cPD: confirmed progressive disease; PD: progressive disease.

Figure 4 shows the outcome according to best iRECIST response in terms of TTP, best tumor shrinkage and CSS. Patients with PR/CR and psPD display similar best tumor shrinkage. Best tumor shrinkage was significantly higher in both subgroups compared to patients with SD or PD as best response or in patients displaying MR evolving toward cPD. Patients with PR/CR and psPD display best TTP and CSS. psPD patients had the second best TTP and CSS, slightly lower compared to TTP and CSS in patients with PR without a phase of psPD and better than TTP and CSS in patients with SD as best response.

### Features associated with psPD

Patient and tumor characteristics in psPD and real PD patients were compared (Table 2). A statistically significant difference was observed in IMDC risk stratification. Globally, IMDC risk prognosis was more favorable in psPD patients compared to patients with real progression. Not any patient classified within the IMDC poor-risk group developed psPD. Although the remaining parameters did not show any statistical significant difference, possibly due to the limited number of patients, several numerical differences were observed. Globally, psPD patients showed more favorable prognostic markers. Patients with psPD were more likely to present with metachronous metastases (54 vs. 29% for patients with real progression) and demonstrated a higher median time to metastasis (37 vs. 13 months). Baseline CRP levels tended to be lower in patients with psPD. In contrast, sarcomatoid dedifferentiation, correlated to aggressive disease, was present in 40% patients with psPD compared to 14% of patients with real progression. These findings are only hypothesis generating due to small patient numbers and a lack of statistical significance of most observations. Moreover, it was impossible to check if patients with psPD experienced improvement of disease-related symptoms at the moment of detection of psPD, because most of the patients were asymptomatic at start of first-line ipilimumab/nivolumab.

**Table 2:** A comparison of patients’ characteristics between patients with pseudoprogression and with real progression and between patients with pseudoprogression and partial response without pseudoprogression.

Patients’ characteristics		psPD (*n* = 13)	Real progression (*n* = 28)	*P*	PR without psPD (*n* = 33)	*P*
Synchronous metastases, *n* (%)		6/13 (46%)	20/28 (71%)	0.17*	15/33 (45%)	1.0*
Median time to metastasis if metachronous, months (range)		27 (3–77)	13 (4–78)	0.94**	23.5 (5–253)	0.6**
Interval < 12 months between diagnosis and start systemic therapy, *n* (%)		8/13 (62%)	19/28 (68%)	0.73*	15/33 (45%)	0.5*
IMDC risk group, *n* (%)	Good	**2/13 (15%)**	**1/28 (3.6%)**	0.03***	7/33 (21%)	0.3***
	Intermediate	**11/13 (85%)**	**17/28 (60.7%)**		22/33 (67%)	
	Poor	**0/13 (0%)**	**10/28 (35.7%)**		4/33 (12%)	
ECOG PS	0	11/13 (85%)	16/28 (57%)	0.16*	29/33 (89%)	1.0*
	1	2/13 (15%)	12/28 (43%)		4/33 (12%)	
Sarcomatoid dedifferentiation present		4/10 (40%)	2/14 (14%)	0.19*	5/24 (21%)	0.4*
Sarcomatoid dedifferentiation mean percentage compared to total tumor volume		8%	2%	0.11^#^	7%	0.3^#^
Median baseline CRP levels (mg/L)		6.9	17.5	0.15^#^	6.6	0.6^#^
Tumor burden (median)(mm)		57	75	0.2^#^	54	0.89^#^

psPD: pseudoprogression; IMDC: International Metastatic Renal Cell Carcinoma Database Consortium; CRP: C-reactive protein; ECOG PS: Eastern Cooperative Oncology Group Performance Status; PR: partial response.

* Fisher’s exact test.

** Kaplan–Meier and log-rank.

*** Chi-square.

# Student test. Significant changes are in bold.

Next, we compared patient and tumor characteristics between patients with psPD and patients with a PR without passing through psPD. Globally, patient and tumor characteristics of patients with PR without passing through psPD were rather similar to characteristics of patients with psPD.

**Figure 5:**
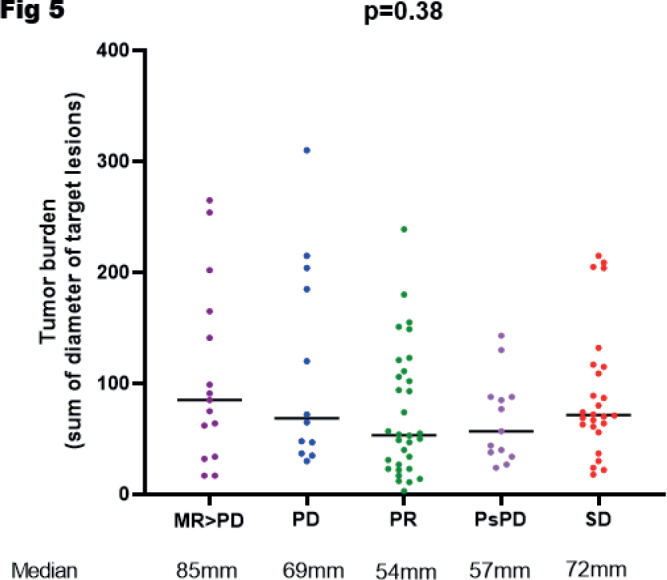
Baseline tumor burden in millimeters of each patient depending on the final response definition. psPD: pseudoprogression; PR: partial response; SD: stable disease; MR: mixed response; PD: progressive disease.

Finally, we compared tumor burden, a factor associated with poorer outcome on ICIs, with response groups (Figure 5). Median tumor burden was the lowest in patients displaying PR and PsPD and the highest in patients with MR evolving to cPD.

## Discussion and conclusions

In m-ccRCC and other tumor types, ICI therapy can lead to atypical responses such as psPD and MR, rendering initial evaluation of progressive lesions more difficult and possibly leading to equivocal therapeutic decisions in patients who could still benefit from continued treatment [13, 14]. Data on psPD and MR in m-ccRCC patients treated with first-line ipilimumab/nivolumab are lacking. We aimed to study the incidence of atypical response patterns in m-ccRCC patients treated with ipilimumab/nivolumab in first-line and impact on outcome.

### A summary of our findings

After correction for atypical responses by iRECIST, on 100 evaluable m-ccRCC patients treated with ipilimumab/nivolumab, MR occurred in 24% of patients. In 62% of these patients, the MR evolved to cPD. In 38% of these patients, the MR evolved toward a PR and was thus classified as a psPD. psPD occurred in 13% of patients. psPD patients had slightly inferior TTP and CSS compared to patients displaying PR as best response without a phase of psPD, but superior TTP and CSS compared to patients with SD as best response. Importantly, we observed that psPD and MR did not only occur at CT1 but also at CT2.

From all patients who presented with RECIST PD at CT1, including 17 patients with uPD and 23 patients with MR, in 12 (30%) patients this was a psPD and led to a PR or CR. Hence, when a first CT scan evaluation shows uPD or MR, the patient still had three chances out of 10 to evolve toward a PR. From all the patients who eventually displayed PR/CR as best response (*n* = 46) after correction for atypical responses by iRECIST, 13 (28%) passed through psPD.

### Comparison with our findings on atypical responses on nivolumab

Our research team recently published a study on atypical responses in 94 m-ccRCC patients treated with nivolumab as a monotherapy in second or further line [17]. The total incidence of psPD and MR were 8.5 and 11.7%, respectively. From the patients with MR, 73% evolved to cPD and 27% to psPD. One out of 7 (14%) of patients with PD at CT1 according to RECIST still evolved toward a PR or long-lasting SD. PFS and OS of patients with psPD were as good as PFS and OS of patients with PR without passing through psPD.

Note that the incidence of psPD (13 vs. 8.5%) and MR (24 vs. 11.7%) and the number of patients with PD at CT1 according to RECIST evolving favorably (30 vs. 14%) is numerically higher in patients treated with ipilimumab/nivolumab as compared to nivolumab in monotherapy, which could be a consequence of the higher efficacy of immunotherapy doublet compared to monotherapy.

### Comparison with literature

In our previous publication, we performed an extensive literature review on the incidence and impact of psPD in m-ccRCC and other tumor types [19]. For the other tumor types, we refer to our previous publication. Here, we only summarize the findings in m-ccRCC that are important for benchmarking of the results of this study. Note that no data are available on psPD in m-ccRCC patients treated with ipilimumab/nivolumab, data are only in patients treated with nivolumab.

In the nivolumab pivotal trial (Checkmate-025), 66 patients were treated beyond progression after PD as best response pre-progression. Nine patients (14%) evolved toward a ≥ 30% tumor burden reduction upon treatment beyond progression. Similar to our series, where IMDC risk groups were scored, patients who responded to treatment beyond first progression, had more often a favorable Memorial Sloan Kettering Cancer Center risk score (55 vs. 35%) [21].

In a retrospective series of 36 m-ccRCC patients treated beyond progression with nivolumab using the newer iRECIST criteria, responses post-uPD were 3 PR (8.3%), 14 SD (39%) and 17 iCPD (47%). Thus, 17/36 (47%) patients had a clinical benefit with nivolumab post-uPD, with a better OS than those without clinical benefit (31.8 months vs. 24.9 months, *p* = 0.02) [22].

Yilmaz found a 11% of psPD in their cohort of 36 m-ccRCC patients treated with nivolumab. All of these patients evolved to a PR or CR after uPD [23].

Finally, in the nivolumab phase II trial (Checkmate-010), 36 mRCC patients were treated beyond progression, leading to 5 PRs (14%) en 21 SD (58%) [24].

Recently, Dinkel et al. published real world data on 356 mRCC patients from Germany and Switzerland treated in first-line with ipilimumab/nivolumab. They report 5% of MR, but correlation with PFS and OS was not reported [25]. This reported MR incidence is substantially lower than in our series. The difference might be explained by the fact that we reanalyzed all CTs with the specific aim of detecting psPD and MR. However, the high MR incidence in our patients should be validated in independent patient series.

### Weaknesses of our study

Our study has several weaknesses. Since this was a retrospective analysis, radiographic evaluations were carried out according to the applicable clinical standard-of-care at the time of treatment. As such, subsequent CT was not always carried out exactly 4–8 weeks after uPD as suggested by the iRECIST guidelines. We also only used the iRECIST and no other available criteria for the evaluation of patients under ICI, such as irRC and irRECIST. However, iRECIST is considered to be the most practical to use in routine practice and studies have shown high concordance between the criteria [17, 18]. Clinical improvement, which would occur in patients with psPD and not in patients with real progression, could not be objectivated. Finally, we did not assess hyperprogression, a separate response type, which can occur under ICI treatment involving altered (increased) tumor growth, possibly due to alteration of the immune system with increased regulatory T-cells, modulation of tumor-promoting cells and aberrant inflammation patterns [26]. Retrospective analyses have described the frequency as 1–7% for m-ccRCC [27].

### Clinical applications

The present data give a precise idea on the incidence of psPD and clinical outcomes after psPD in m-ccRCC treated with ipilimumab/nivolumab and hence support clinicians to treat beyond progression on CT1.

To detect in advance who will evolve from MR or uPD on CT1 to cPD or to PR remains a challenge. Patients with psPD usually displayed more favorable disease and tumor characteristics compared to patients with real PD: they were more often IMDC good risk and tend to present more often with metachronous metastases, higher median time to metastasis and lower baseline CRP levels. Their tumors displayed more often sarcomatoid dedifferentiation. Hence, the probability of favorable outcome in case of treatment continuation in the presence of uPD or MR at CT1 might be higher in patients and diseases with these characteristics. However these findings are only hypothesis generating, and treatment continuation beyond uPD or MR at CT1 remains standard of care, also in more aggressive m-ccRCC and in tumors without sarcomatoid dedifferentiation. Only in case of very important disease progression at CT1 with a risk of organ failure, early switch to second-line VEGFR-TKI therapy can be indicated. Improvement of clinical symptoms at CT1 can announce psPD, while clinical deterioration at CT1 can announce real progression.

In case of oligoprogression in later timepoints of ipilimumab/nivolumab therapy, metastasis directed therapy (surgery or radiation therapy) on the progressing lesion can also be indicated while systemic therapy is continued. Radiation therapy on a progressing lesion on CT1 is usually not preferred, but can be indicated for symptom control.

In summary, our study reports for the first time the incidence of atypical responses such as psPD (13%) and MR (24%) in m-ccRCC patients treated with ipilimumab/nivolumab in m-ccRCC. While psPD is correlated with favorable outcome, MR can evolve to favorable outcome (38%) or can be the first sign of progression (62% of cases). Three patients out of 10 (30%) who display uPD or MR at first CT-evaluation, still evolved favorably toward a PR. Although clinicians are now aware of the potential for psPD and the recommendation to continue ICIs beyond uPD, our study provides more precise estimates of its incidence in m-ccRCC patients treated with ipilimumab/nivolumab.

## Data Availability

Data are available upon request by sending an e-mail to the corresponding author.
